# Correction to: “Obesity and contraceptive use: impact on cardiovascular risk”

**DOI:** 10.1002/ehf2.14322

**Published:** 2023-02-27

Funding section was not complete.

This should have read: “This study was supported by the Italian Ministry of Health (Ricerca Corrente) 20/1904”.

We apologize for this error.

